# Impact of anthropometrical parameters on portal vein diameter and liver size in a subset of Karachi based population

**Published:** 2014

**Authors:** Tanya Raza Siddiqui, Nuzhat Hassan, Pashmina Gul

**Affiliations:** 1 Dr. Tanya Raza Siddiqui, MBBS, Senior Lecturer, Department of Anatomy, Ziauddin College of Medicine, Ziauddin University Hospital, Clifton Campus,Karachi, Pakistan.; 2 Prof. Nuzhat Hassan, M. Phil, Professor and Chairperson, Department of Anatomy, Ziauddin College of Medicine, Ziauddin University Hospital, Clifton Campus,Karachi, Pakistan.; 3 Dr. Pashmina Gul, FCPS, Assistant Professor, Department of Radiology, Ziauddin College of Medicine, Ziauddin University Hospital, Clifton Campus,Karachi, Pakistan.

**Keywords:** Anthropometrical measurements, Liver, Portal vein diameter

## Abstract

***Objective:*** The purpose was to study the impact of anthropometrical parameters on portal vein diameter and liver size by ultrasound in a subset of Karachi population.

***Methods:*** Four hundred and fifty nine apparently healthy subjects were included in this cross sectional study. After recording weight and height of each subject, Portal vein diameter and both liver lobes were measured by gray scale ultrasonography. Students T test and ANOVA were applied for statistical analyses.

***Results:*** With increasing age, portal vein diameter and right lobe of liver increased significantly (p value < 0.001). Increase in portal vein diameter was also observed with rise in body mass index (0.8 cm in underweight - 1.1 cm in obese subjects). Sizes of right and left liver lobes also increased with a rise in body mass index (p value < 0.001 and 0.001). Gender, however, did not have any effect on portal vein diameter and liver size.

***Conclusion:*** Age and body mass index are reliable parameters to consider for avoiding false positive diagnosis of hepatomegaly and portal hypertension. Knowing the right and left liver size with respect to anthropometrical measurements also assist a clinician in selecting a subject for liver transplantation.

## INTRODUCTION

Liver is the largest organ of the body.^1^ It encompasses functions pertaining to complex biochemical and metabolic pathways.^1^ Previous studies have reported that clinical evaluation of liver size is an important prerequisite to avoid liver failure and small-for-size graft syndrome in liver transplantation.^2^ Several studies have demonstrated that liver size depends upon several factors: age, gender, nutrition and body surface area.^1^^,^^3^^-^^5^ Furthermore, establishing the hepatic span by assessing these physical parameters would improve the accuracy of clinical assessment in a particular population according to its own geographic genome.^1^^,^^4^^-^^6^ Anatomically, portal vein (PV) is intricately related to the liver architecture because it carries deoxygenated but nutrient rich blood from the gastrointestinal tract towards liver in a hepatopetal flow.^3^^,^^6^^,^^7^ Any pathology in the hepatic vasculature and parenchyma would eventually affect the diameter and flow of the PV leading to cirrhosis and portal hypertension.^4^^,^^8^

In past decade, extensive work has been conducted in different parts of the world on portal vein diameter (PVD).^3^^-^^5^^,^^9^ However, no specific data has been reported in our part of the world on the effect of different physical parameters on PVD and hepatic span.^10^^,^^11^ In an Asian hepatitis survey, Pakistan has been reported to be at a high risk of hepatitis C infection which can eventually lead to liver failure.^12^ Karachi, the largest cosmopolitan city of Pakistan, has an alarming rise in hepatitis B and C infection and its complications.^13^ Reasons are attributed to a monumental influx of immigrants, lack of awareness and inadequacy of blood screening.^13^^,^^14^

This study was designed keeping in mind the vast spectrum of hepatobiliary diseases which can affect any age group. Also, in invasive procedures like liver transplantation, trans-hepatic portal vein embolization and pancreatectomy the portal vein is anastomosed with other vessels. Therefore, knowing the normal caliber of the vein and the hepatic span, with respect to age, gender and body mass index (BMI) is crucial for diagnosis of portal hypertension and hepatomegaly.

## METHODS

In this cross sectional study, four hundred and fifty nine healthy adults who met the inclusion criteria were recruited at Radiology clinic of Ziauddin University Hospital (Clifton campus) over a period of eight months. It was a convenience sampling in which the paramedical staff of Ziauddin Hospital had participated after its announcement. Subjects with hypertension, diabetes, hepatobiliary diseases, myloproliferative disorders, cardiac diseases and pregnant females were excluded. This study was approved by ethical review committee of Ziauddin University. Medical history and personal data (blood pressure, weight, height and age) was recorded before sending the subject for ultrasound. Subjects gave written consent and were allotted serial numbers to safe guard their identity.

The gray scale sonographical examination was performed with a high-resolution real-time scanner (Toshiba version, Nemio XG) with a 3.75 MHz convex curved array transducer. Subjects were requested to report in the morning after an overnight fast as certain type of foods may affect the diameter of portal vein as suggested by Kok et al.^15^ The measurements of dimensions of the liver size were made in left lateral decubitus position during deep inspiration. Longitudinal scans of right lobe (R-lobe) were obtained from the subcostal approach in the midclavicular position and left lobe (L-lobe) from the anterior subxiphoid approach. The diameter of the extra hepatic part of the portal vein was measured at the level of the porta hepatis where visualization was optimal. Both liver and portal vein were measured three times before the mean value was recorded by a single sonographer to avoid inter-observer variations.

Classification of PVD and liver lobes was done on the basis of age, BMI and gender. Subjects were divided into three groups according to age i.e.; 20-30 years (group 1), 31-40 years (group 2) and 41 years and above (group 3). BMI classification was done as suggested by World Health Organization (WHO) in the Asian population. Subjects with BMI less than 18.50 were classified as underweight. Those with BMI between 18.50- 24.99 were taken as normal weight. An individual with BMI ranging from 25.00-29.99 was classified as overweight and if BMI was 30.00 or more, it was in the obese bracket. On the basis of gender two groups of male and females were organized.

Data was entered and analyzed using statistical software SPSS version 20. Mean±SD was computed for the diameter of portal vein and sizes of right & left lobe of liver. Student’s T-test was applied to compare continuous variables across gender. ANOVA was applied for comparison across continuous variables (PVD and liver span) for age and BMI among genders. Results with p-value less than 0.05 were considered as statistically significant.

## RESULTS

In our study liver span and PVD were compared in different age groups. The diameter of PV in a subset of Karachi based population varies with increasing age as shown in Table-I. As the age was increasing PVD and the right liver lobe increased with significant P value of < 0.001.

With respect to BMI, there was significant difference also. The PVD increased from 0.8cm in underweight to 1.1cm in obese subjects. Furthermore, p-value was highly significant for both the lobes of liver as shown in Table-II with increasing BMI.

Table-III depicts effects of gender. The mean age of males was 35.7+11.3 years and in females it was 34+11.3 years. There was no significant statistical difference between any parameter.

## DISCUSSION

**Fig.1:**
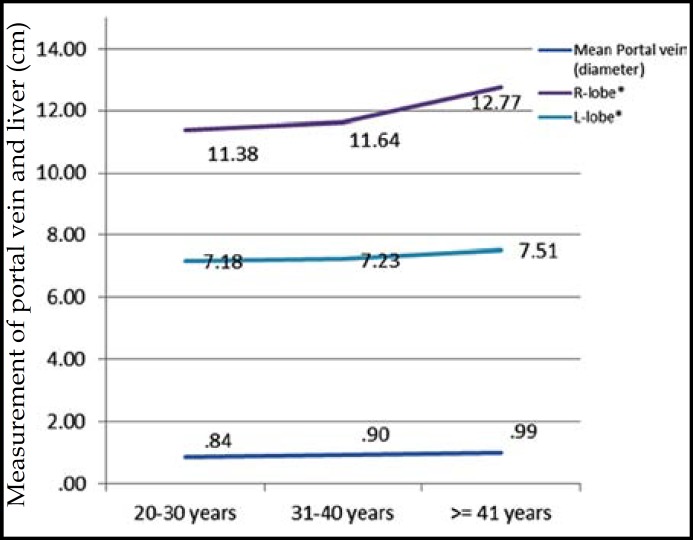
Impact of increasing age on portal vein and liver lobes

There is abundant data in different parts of the world on the normal and diseased hepatobiliary system which includes autopsies also.^16^^-^^23^ Kratzer et al. conducted a massive sonographical survey concluding that age had a strong influence on hepatic span similar to our study as shown in Fig.1.^24^ Similarly, Udoh et al also suggested that liver size of 14.20+1.62 cm does not point towards hepatomegaly in Nigerians and as the age advances the liver size also increases.^25^ Increase in the liver size is attributed to build up in the work load and physiological adaptation for rise in metabolic demands with advancing age.^26^ In our study, we measured the right and left lobes separately because it was noted by Ho CM et al that liver diseases usually occur in the right lobe because of the composition of blood that supplies it.^27^ The liver size obtained by Mittal et al in Rajasthan (India) was comparatively higher than that observed in our study.^28^ This is probably due to racial differences. In contrast to the above studies, colleagues of Tetsuya and Chouker et al who have studied liver sizes with respect to age have suggested that the liver size decreases with age.^2^^,^^29^

**Table-I:** Relationship of portal vein diameter and liver span with increasing age

	*Group 1* *20-30 years (N=159)*	*Group 2* *31-40 years (N=199)*	*Group 3* *> 41 years (N=101)*	*p-value*
PVD (cm)	0.84+0.14	0.90+0.17	0.99+0.21	<0.001
R- LOBE SPAN (cm)	11.38+1.67	11.64+1.80	12.77+1.51	<0.001
L-LOBE SPAN (cm)	7.18+2.12	7.23+2.06	7.51+2.53	0.45

**Table-II:** Relationship of portal vein and liver span with increasing BMI

	*Under Weight (N=41)*	*Normal Weight (N=314)*	*Over Weight (N=86)*	*Obese (N=18)*	*P-Value*
PVD (cm)	0.8+0.2	0.9+0.2	0.9+0.2	1.1 +0.4	0.005
R-LOBE SPAN (cm)	9.4+1.2	11.5+1.5	13.4+0.9	13.9+0.7	<0.001
L-LOBE SPAN (cm)	6.4+2.2	7.2+2.2	7.7+1.9	7.8+2.5	0.01

**Table-III:** Relationship of portal vein and liver span with gender

	*Male (N= 231)*	*Female (N=228)*	*p-value*
PVD (cm)	0.9+0.2	0.9+0.2	0.38
R-LOBE SPAN (cm)	11.9+1.7	11.7+1.9	0.412
L-LOBE SPAN (cm)	7.1+2.2	7.4+2.2	0.146

*PVD: portal vein diameter, R-LOBE SPAN: right lobe of liver,*

*L-LOBE SPAN: left lobe of liver. BMI: body mass index.*

Our study investigated effects of BMI on the liver span to provide a guideline for endocrinologists to prevent nonalcoholic fatty liver disease (NAFLD). It has been reported that weight gain and obesity are one of the major causes for developing NAFLD.^30^ A limitation of our study was that in the obese bracket only 18 subjects could be enrolled because many had to be excluded because of asymptomatic fatty liver.

A novel study conducted in Jordan suggested that body surface area in females and height in males were suitable physical parameters to assess liver span.^31^ Our results support Kratzer et al in concluding that BMI is an important parameter while measuring the extent of liver size.^24^

Literature search has revealed contrasting measurements of portal vein diameter in different countries which maybe because of diverse genome and technical differences.^4^^,^^5^^,^^9^^,^^32^^,^^33^ Covey et al suggested that the understanding of PV anatomy is important for interventional radiologists and surgeons.^34^ Our results showed significant positive correlation between PVD and age which are in agreement with Shankar et al who also concluded that with increasing age PVD also increases.^3 ^However, the author had neglected to mention whether the measurements were pre or post prandial. Enlargement of PVD with increasing age may be explained by fragmentation of smooth muscles and loss of elasticity in the reticular network.^35^ Jeffery et al hypothesized that a caliber greater than 1.3 cm would point towards portal hypertension.^36^ However, the validity of this statement needs to be tested in a larger sample size and in comparison to patients with portal hypertension.

Our results of table 3 are compatible with Kratzer et al in concluding that gender did not affect the PVD and liver size.^24^ A Doppler ultrasound study done in Iran on a small population concluded that gender was an important physical aspect for the determination of PVD.^5^ Udoh et al have also suggested that gender is an important factor to influence the liver span.^25^ Nonetheless, BMI was not taken into consideration which could have further validated these results.

Our study has concluded that age and BMI are important physical parameters that affect PVD and liver span. Results of this study can be considered in diagnosing NAFLD and portal hypertension. This reference range of our population can also be applied in routine radiological clinics. Future work should include large scale nationwide studies which can guide a clinican in selecting subjects for liver transplantation and for further strengthening our results. 

## Notes

***Conflict of interest & funding: ***No conflict of interest or funding resources to declare.

## Notes

***Authors contribution:- ***The concept, data collection and its analysis was done by Dr. Tanya Raza Siddiqui. Dr. Nuzhat Hassan critically analyzed and gave the final approval. Dr. Pashmina Gul provided the clinical input.

## References

[1] Wolf DC, Walker HK, Hall WD, Hurst JW, Chapter 94 (1990). Evaluation of size, shape and consistency of liver. Clinical Methods: The History, Physical and Laboratory Examinations.

[2] Kiuchi T, Oike F, Yamamoto H (2003). Small-for-size graft in liver transplantation. Nagoya J Med Sci.

[3] Shankar R, Shetty GS, Srinath MG, Kulkarni R (2011). Estimation of Portal Vein Diameter in co – Relation with the Age, Sex and Height of An Individual. Anatomica Karnataka.

[4] Anakwue AC, Anakwue RC, Ugwu AC (2009). Sonographic Evaluation of Normal Portal Vein Diameter in Nigerians. Euro J Sci Res.

[5] Yazdi HR, Sotoudeh H (2006). Assessment of Normal Doppler Parameters of Portal Vein and Hepatic Artery in 37 Healthy Iranian Volunteers. Iran J Radiol.

[6] Ozbulbul NI (2011). Congenital and acquired abnormalities of the portal venous system: multi detector CT appearances. Diagn Interv Radiol.

[7] Al-Nakshabandi NA (2006). The role of ultrasonography in portal hypertension. Saudi J Gastroenterol.

[8] Webster, Burroughs, Riordan (2005 Jan 1). Portal vein thrombosis – new insights into aetiology and management. Aliment Pharmacol Ther.

[9] Hawaz Y, Admassie D, Kebede T (2012). Ultrasound Assessment of Normal Portal Vein Diameter in Ethiopians Done at Tikur Anbessa Specialized Hospital. East Cent Afr J Surg.

[10] Kamal MM, Niazi M, Umar M (2009). Sensitivity and Specificity of Ultrasonography in the Early Diagnosis of Liver Fibrosis Stage in Patients with Chronic Liver Disease. Ann Pak Inst Med Sci.

[11] Siddiqui EH, Siddiqui S, Shah N (2012). Ultrasound; evaluation of hepatobiliary system: a local perspective. Professional Med J.

[12] Sievert, Altraif, Razaavi et al A systemic review of hepatitis C virus epidemiology in Asia, Australia and Egypt. Liver International.

[13] H.Nazar, N.Nadia, N. Shazia (2008). Prevalence of Hepatitis B and C in blood donors of Karachi. Biomedica.

[14] Muhammad Jamil, Hammad Ali, Robina Shaheen, Abdul Basit (2010). Prevalence, Knowledge and Awareness of Hepatitis C among Residents of Three Union Councils in Mansehra. J Ayub Med Coll Abbottabad.

[15] Koc Z, Oguzkurt L, Ulusan S (2007). Portal vein variations: clinical implications and frequencies in routine abdominal multidetector CT. Diagn Interv Radiol.

[16] HS.Brown, M. Halliwell, M. Qamar, A.E. Read, J M Evans, P.N.T Well (1989). Liver, Biliary and Pancreas. Measurement of Normal Portal Venous Blood Flow by Doppler Ultrasound. Gut..

[17] Ulusan S, Yakar T, Koc Z (2011). Evaluation of portal venous velocity with Doppler ultrasound in patients with nonalcoholic fatty liver disease. Korean J Radiol.

[18] Lopa Mudra Mandal, Kumar Mandal, Dipanjan Bandyopadhyay, Saumik Datta (2011). Correlation of Portal Vein Diameter and Splenic Size with Gastro-oesophageal Varices in Cirrhosis of Liver. JIACM.

[19] Siddiqui TR, Hassan N, Gul P (2013). Effect of anthropometrical measurements on portal vein and hepatosplenic span. Pak J Med Sci.

[20] Shateri K, Mohammadi A, Moloudi F, Nosair E, Ghasemi-Rad M (2012). Correlation Between Sonographic Portal Vein Diameter and Flow Velocity With the Clinical Scoring Systems MELD and CTP in Cirrhotic Patients: Is There a Relationship?. Gastroenterol Res.

[21] Christina Tziafalia, Marianna Vlychou, Konstantina Tepetes, Nikoloas Kelekis, Ioannis Fezoulidis (2006). Echo-Doppler Measurements of Portal Vein and Hepatic Artery in Asymptomatic Patients with Hepatitis B Virus and Healthy Adults. J Gastrointestin Liver Dis December.

[22] Rahim N, Adam EJ (1985). Ultrasound demonstration of variations in normal portal vein diameter with posture. Br J Radiol.

[23] Chuo LS, Mahmud R, Salih QA (2005). Color Doppler Ultrasound Examination of the Main Portal Vein and Inferior Vena Cava in Normal Malaysian Adult Population: A Fasting and Post Prandial Evaluation. Internet J Cardiovascular Res.

[24] Kratzer W, Fritz V, Mason RA, Haenle MM, Kaechele V (2003). Factors Affecting Liver Size, A Sonographic Survey of 2080 Subjects. J Ultrasound Med.

[25] Udoh BE, Eze JC, Chiegwu HU (2011). Sonographic Assessment of Liver Sizes in Healthy South East Nigerians. Am J Sci Res.

[26] David Andrew (26th May 200s). Interpretation of Liver Enlargement in Regulatory Toxicity Studies. PSD Guidance document.

[27] Ho CM, Lin RK, Tsai SF et al (2010 Apr). Simulation of portal hemodynamic changes in a donor after right hepatectomy. J Biochem Eng.

[28] Mittal R, Chowdhary DS (2010). A Pilot Study of the Normal Measurements of the Liver and Spleen by Ultrasonography in the Rajasthani Population. JCDR.

[29] Alexander Chouker, Andre Martignoni, Martin Dugas, Wolfgang Eisenmenger, Rolf Schauer, Ines Kaufmann et al (2004). Estimation of Liver Size for Liver Transplantation: The Impact of Age and Gender. Liver Transplantation.

[30] Wree A, Kahraman A, Gerken G, Canbay A (2010). Obesity Affects the Liver – The Link between Adipocytes and Hepatocytes. Digestion.

[31] Tarawneh ES, Hadidy AM, Haroun AA, Mahafza WS, Samara OA, Arafeh FM (2009). Ultrasound Measurement of Liver Span in Jordanian Adults: A Preliminary Experience. J Med J.

[32] Patriquin, Lafortune, Burns et al (1987). Duplex Doppler Examination in Portal Hypertension: Technique and Anatomy. AJR.

[33] Andrew K Burroughs, James S. Dooley, Anna S.F. Lok, Andrew K. Burroughs, E. Jenny Heathcote (2011). The Hepatic Artery, Portal Venius System and Portal Hypertension: the hepatic veins and liver in circulatory Failure. Sherlock’s Diseases of the Liver and Biliary System.

[34] Covey AM, Brody LA, Getrajdman GI, Sofocleous CT, Brown KT (2004). Incidence, patterns, and clinical relevance of variant portal vein anatomy. AJR Am J Roentgenol.

[35] Adibi A, Givechian B (2007). Diameter of common bile duct: what are the predicting factors?. JRMS.

[36] Jeffery Weinreb, Sheila Kumari, Gail Philips ( 1982). Portal vein measurements by real time sonography. The American Journal of Radiology. AJR.

